# The Ventilatory Ratio as a Predictor of Successful Weaning from a Veno-Venous Extracorporeal Membrane Oxygenator

**DOI:** 10.3390/jcm13133758

**Published:** 2024-06-27

**Authors:** Anna Fischbach, Steffen B. Wiegand, Julia Alexandra Simons, Liselotte Ammon, Rüdger Kopp, Guillermo Ignacio Soccoro Matos, Julio Javier Baigorri, Jerome C. Crowley, Aranya Bagchi

**Affiliations:** 1 Department of Anesthesiology, University Hospital RWTH Aachen, Pauwelsstraße 30, 52074 Aachen, Germany; 2 Department of Anesthesiology and Intensive Care Medicine, Hannover Medical School, 30625 Hannover, Germany; 3 Department of Operative Intensive Care Medicine, University Hospital RWTH Aachen, Pauwelsstraße 30, 52074 Aachen, Germany; 4 Department of Anesthesiology, Massachusetts General Hospital, Boston, MA 02114, USA

**Keywords:** ARDS, ventilatory ratio, extracorporeal membrane oxygenation, weaning

## Abstract

**Background:** Veno-venous extracorporeal membrane oxygenation (VV-ECMO) is a critical intervention for patients with severe lung failure, especially acute respiratory distress syndrome (ARDS). The weaning process from ECMO relies largely on expert opinion due to a lack of evidence-based guidelines. The ventilatory ratio (VR), which correlates with dead space and mortality in ARDS, is calculated as [minute ventilation (mL/min) x arterial pCO_2_ (mmHg)]/[predicted body weight × 100 × 37.5]. **Objectives:** The aim of this study was to determine whether the VR alone can serve as a reliable predictor of safe or unsafe liberation from VV-ECMO in critically ill patients. **Methods:** A multicenter retrospective analysis was conducted, involving ARDS patients undergoing VV-ECMO weaning at Massachusetts General Hospital (January 2016 – December 2020) and at the University Hospital Aachen (January 2012–December 2021). Safe liberation was defined as no need for ECMO recannulation within 48 h after decannulation. Clinical parameters were obtained for both centers at the same time point: 30 min after the start of the SGOT (sweep gas off trial). **Results:** Of the patients studied, 83.3% (70/84) were successfully weaned from VV-ECMO. The VR emerged as a significant predictor of unsafe liberation (OR per unit increase: 0.38; CI: 0.17–0.81; *p* = 0.01). Patients who could not be safely liberated had longer ICU and hospital stays, with a trend towards higher mortality (38% vs. 13%; *p* = 0.05). **Conclusions:** The VR may be a valuable predictor for safe liberation from VV-ECMO in ARDS patients, with higher VR values associated with an elevated risk of unsuccessful weaning and adverse clinical outcomes.

## 1. Introduction

The benefit of veno-venous extracorporeal membrane oxygenation (VV-ECMO) is to allow oxygenation and to remove CO_2_ while lung protective strategies are carried out to reduce ventilator-induced lung injury [1]. Indications of VV-ECMO involve cases with severe respiratory failure, such as acute respiratory distress syndrome (ARDS) where the mortality risk exceeds 50% even with optimal conventional therapies [2]. Once the lungs of the patient have recovered, a weaning trial can be initiated. Nowadays, weaning strategies are based on the opinion of experts rather than evidence [3,4], and weaning protocols differ widely [1,3,5,6,7,8,9,10,11,12,13,14,15,16]. Detailed ECMO weaning strategies and specific predictors of ECMO weaning success are lacking [5]. Increased total dead space is frequently observed in cases with ARDS [17,18] and is associated with increased mortality. Increased dead space also reduces the likelihood of successful decannulation from VV-ECMO. However, the measurement of dead space can be very challenging due to the requirement for specialized equipment [19,20]. In 2009, the ventilatory ratio (VR) was introduced as a simple bedside measure of ventilation [21]. Further studies showed that the VR correlates positively with measured dead space [22] and is also correlated with higher mortality in patients with ARDS [20,22,23,24].

The VR is defined as [minute ventilation (mL/min) × arterial pCO_2_ (mmHg)]/[predicted body weight × 100 × 37.5], whereas the predicted body weight can be calculated using the formula 50 + 2.3 × (height (inches) − 60) for males, and 45.5 + 2.3 × (height (inches) − 60) for females, respectively [25].

We hypothesized that patients who were successfully decannulated from VV-ECMO had a lower VR compared to those who were not successfully decannulated when the VR was measured after the sweep gas circuit was capped. Therefore, the aim of this retrospective study was to assess the efficacy of the VR as a tool to predict successful weaning in patients treated with VV-ECMO.

## 2. Materials and Methods

We conducted a multicenter retrospective study including the Massachusetts General Hospital in Boston in the US (patients were included from January 2016 to December 2020) and the University Hospital Aachen in Germany (patients were included from January 2012 to December 2021). This study followed the analytical approach that was previously described by Al-Fares [7]. The study was approved by the IRB from MGH and the ethics committee of the University Hospital Aachen (2024P000698, date of approval 25th January 2024 and EK223-22, date of approval 21st June 2022), including adults (aged ≥ 18 years) diagnosed with ARDS, connected and weaned from VV-ECMO. Patients were excluded if they (1) failed to meet the inclusion criteria or if they (2) required multiple attempts at weaning.

### 2.1. Study Population

**Figure 1:**
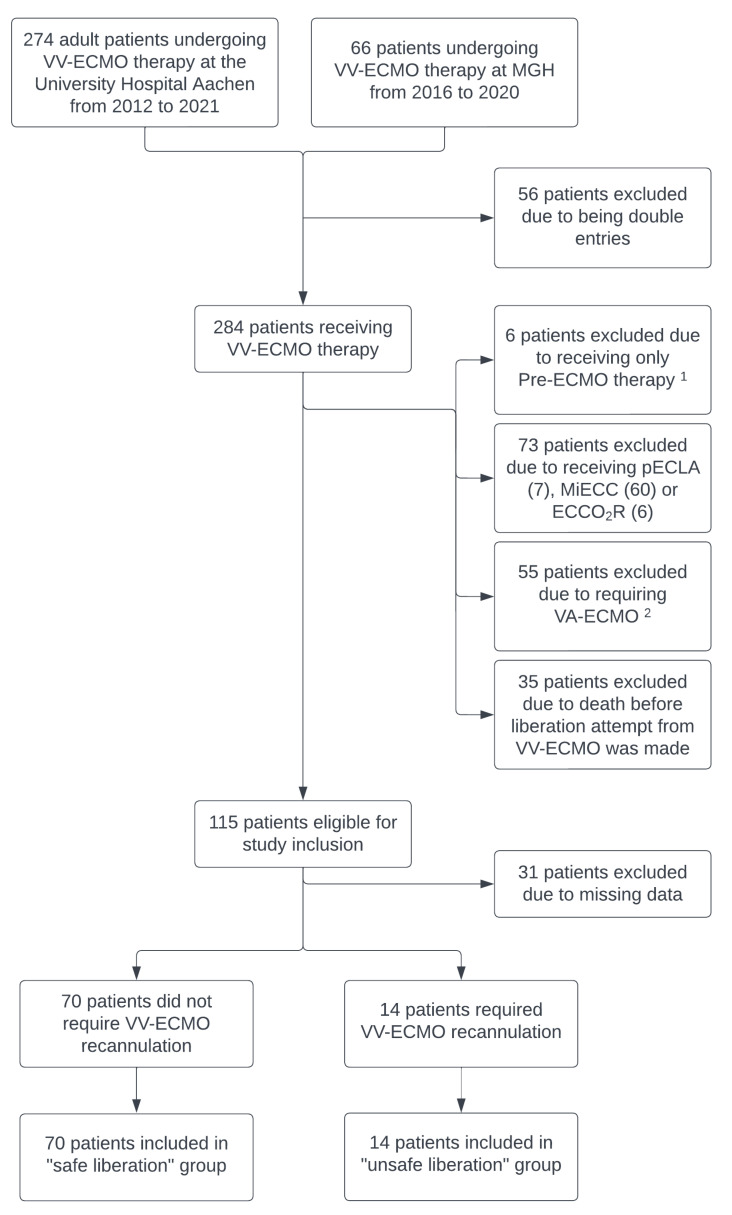
Study flow chart of the study population. VV-ECMO: veno-venous extracorporeal membrane oxygenation; VA-ECMO: veno-arterial extracorporeal membrane oxygenation; pECLA: pumpless extracorporeal lung assistance; MiECC: minimal invasive extracorporeal circulation; ECCO_2_R: extracorporeal CO_2_ removal. ^1^ Pre-ECMO therapy refers to preparations for ECMO that do not proceed to cannulation, often due to patient death before ECMO can be initiated. ^2^ 55 patients initially required only VV-ECMO but were later escalated to VA-ECMO, leading to the exclusion from this study.

Between 2012 and 2021, 274 patients from the University Hospital Aachen and 66 from Massachusetts General Hospital were initially identified for VV-ECMO therapy (Figure 1). After removing duplicates, 284 patients remained. Exclusions were made for 134 patients: 6 only received pre-ECMO therapy, 7 underwent pumpless extracorporeal lung assistance (pECLA), 60 received minimal invasive extracorporeal circulation (MiECC), 6 only had extracorporeal CO_2_ removal (ECCO_2_R), and 55 patients were transitioned from VV-ECMO to VA-ECMO. After the further exclusion of 35 patients with no liberation attempt before death, 115 were eligible for study inclusion. Additionally, 31 patients were excluded for incomplete medical records, leaving 84 patients for study inclusion.

Weaning was performed in accordance with the concurrent strategy at the respective hospital (Appendix A). Clinical parameters were obtained for both centers at the same time point: 30 min after the start of the SGOT (sweep gas off trial). The primary outcome of the study was unsafe liberation from VV-ECMO defined as the absence of VV-ECMO recannulation within 48 h after decannulation.

### 2.2. Statistical Analysis

The statistical analysis was performed using GraphPad Prism software (v.8.3). Normal distribution was tested by using the Shapiro–Wilk test. The baseline characteristics of the patients were compared by using a Mann–Whitney test. Multivariate analyses were performed by using SPSS software Version 22 (SPSS Inc., Chicago, IL, USA). The inclusion of the parameters for the logistic regression model was based on results from the Mann–Whitney test. If *p* < 0.1, the parameter was included. A binary logistic regression analysis was performed; odds ratios and confidence intervals (95%) were calculated for the model. Receiver operating characteristic (ROC) curves were used to illustrate the performance of the VR and PFR as predictor variables for unsafe liberation from VV-ECMO. The Youden index (J) was calculated to find an optimal threshold for the VR and PFR (P/F ratio; p_a_O_2_/FiO_2_) in Excel (v.2311; Microsoft Corp., Redmond, WA, USA) with this function: J = sensitivity + specificity − 1. The outcome analysis was performed by using a log-rank test with hazard ratios calculated with the Mantel–Haenszel method. Kaplan–Meier curves were used to illustrate the analysis. Statistical significance was defined as *p* < 0.05. In all statistical tests performed, all patients were always included. If the inclusion of all patients was not possible due to missing data, this information was explicitly added in the description below the table or figure.

## 3. Results

### 3.1. Baseline Characteristics

Notably, 70 out of 84 (83%) patients could safely be weaned from VV-ECMO, and the patients had a mean age of 51 years (36–60 years). A total of 14 patients (17%) could not be weaned from VV-ECMO (Table 1). The primary cause of ARDS was of pulmonary origin.

Specifically, 36% of all patients were diagnosed with ARDS caused by COVID-19. Among the patients who could be successfully weaned from VV-ECMO, 33% had COVID-19-induced ARDS, while 50% of patients who were unsafely liberated from VV-ECMO had COVID-19-induced ARDS (*p* = 0.36). The clinical characteristics of the patients with a failed weaning attempt were comparable to those who could be successfully weaned from VV-ECMO, except for the duration on VV-ECMO, which was longer in the patients with an unsuccessful weaning attempt (16 days (13–19 days) versus 10 days (4–19 days), *p* = 0.04).

### 3.2. VR and PFR of Patients with Severe ARDS with Safe or Unsafe Liberation

Patients with unsafe liberation from VV-ECMO had a significantly higher VR compared to the patients who could be safely liberated from VV-ECMO (1.89 (1.79–2.81) vs. 1.58 (1.32–1.91); *p* < 0.01) (Table 2). Patients with unsafe liberation from VV-ECMO showed a significantly lower PFR (P/F ratio; p_a_O_2_/FiO_2_) compared to the patients who could be safely liberated from VV-ECMO (169 (110–212) vs. 243 (185–287); *p* = 0.01).

### 3.3. Predictors of Unsafe Liberation from VV-ECMO

A logistic regression was performed to ascertain the effects of the VR, PFR, and duration of VV-ECMO (in days) on the unsafe liberation from VV-ECMO (Supplement S1). The VR, PFR, and duration of VV-ECMO were included because these parameters differed with a *p* < 0.1 in the univariate analysis. The logistic regression model was statistically significant (χ^2^(3) = 9.2; *p* = 0.027). The model explained 17.6% (Nagelkerke *R^2^*) of the variance in unsafe liberation from VVECMO and correctly classified 86.0% of cases. The VR was the only parameter that was shown to be a predictor of unsafe liberation from VV-ECMO (OR: 0.38 (CI: 0.17–0.81; *p* = 0.01) (Table 3). If the VR increased by 1, the probability of successful liberation from VV-ECMO is reduced by 62%.

**Figure 2:**
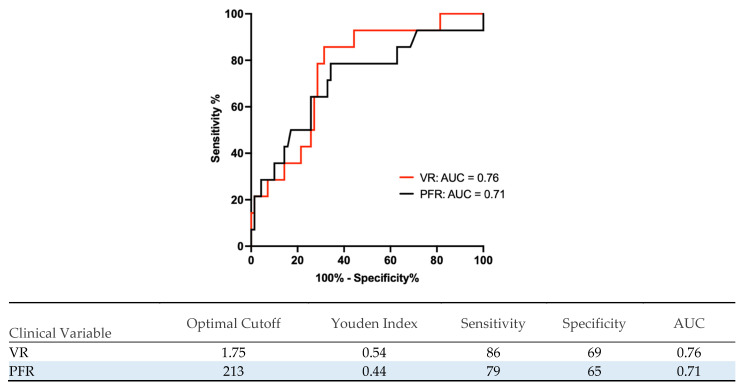
Receiver operating characteristics (ROCs) and AUC for the VR and PFR 30 min after the start of the sweep gas off trial for 70 patients with safe liberation and 14 patients with unsafe liberation from VV-ECMO. Using the receiver operating characteristics (ROCs), we identified the cutoffs with the highest sensitivity for the VR (1.75) and PFR (213). AUC: area under the curve; VR: ventilatory ratio; PFR: P/F ratio (P_a_O_2_/FiO_2_); VV-ECMO: veno-venous ECMO.

In order to illustrate the performance of the VR and PFR, a ROC analysis was performed. The AUC for the VR was 0.76, and for the PFR, it was 0.71. The cutoffs for the VR were determined at 1.75 (sensitivity: 86%, specificity: 69%, J = 0.54) and PFR 213 (sensitivity: 79%, specificity: 65%, J = 0.44) (Figure 2). To reach a sensitivity >90% at the cost of specificity, different cutoffs were determined. The VR cutoff was 1.64 (sensitivity: 93%, specificity: 56%, J = 0.49) and the PFR cutoff was 276 (sensitivity: 93%, specificity: 29%, J = 0.22).

### 3.4. Secondary Outcome

**Figure 3:**
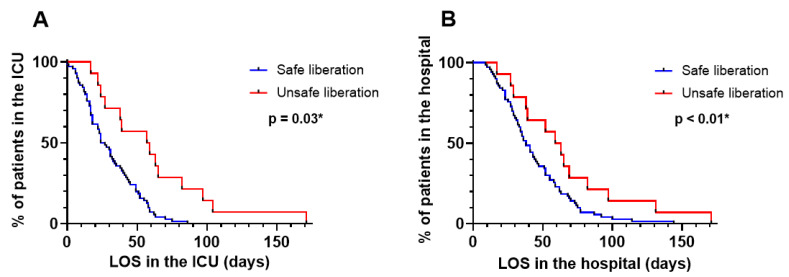
LOS in the ICU and in the hospital for patients with safe and unsafe liberation from VV-ECMO. (**A**) ICU LOS of patients with successful and failed weaning attempts from VV-ECMO. (**B**) Hospital LOS of patients with successful and failed weaning attempts from VV-ECMO. Patients with failed weaning were 2.5 and 1.8 times more likely to have an extended LOS in the ICU and in the hospital. LOS: length of stay; ICU: intensive care unit; VV-ECMO: veno-venous ECMO. Significant results are marked with an *.

**Figure 4:**
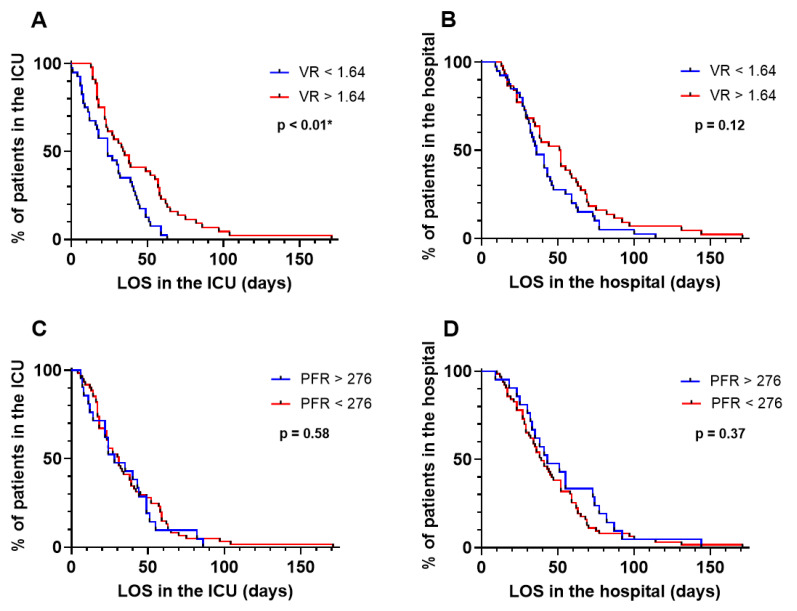
LOS in the ICU and in the hospital for patients with a VR > 1.64 and a VR < 1.64 and with a PFR > 276 and a PFR < 276. (**A**) LOS in the ICU of patients with a VR > 1.64 and a VR < 1.64. (**B**) LOS in the hospital of patients with a VR > 1.64 and a VR < 1.64. Patients with a VR > 1.64 were 2.2 times more likely to have an extended LOS in the ICU compared to those who had a VR < 1.64. There was no significant association between a VR > 1.64 and the LOS in the hospital. LOS: length of stay; ICU: intensive care unit; VR: ventilatory ratio. (**C**) LOS in the ICU of patients with a PFR > 276 and a PFR < 276. (**D**) LOS in the hospital of patients with a PFR > 276 and a PFR < 276. There was no significant association between the PFR and LOS in the ICU or hospital. LOS: length of stay; ICU: intensive care unit; PFR: P/F ratio (P_a_O_2_/FiO_2_). Significant results are marked with an *.

The hospital LOS (61 days (38–79 days) vs. 38 days (27–59 days); *p* = 0.04) and ICU LOS (58 days (30–78 days) vs. 26 days (16–45 days); *p* < 0.01) were significantly higher in the patients unsafely liberated from VV-ECMO (Table 4). There was a trend towards higher mortality between the patients with safe and unsafe liberation from VV-ECMO (13% vs. 38%; *p* = 0.05). Patients with failed weaning were 2.5 and 1.8 times more likely to have an extended LOS in the ICU (HR: 2.5; CI: 1.5–4.1; *p* < 0.01) and in the hospital (HR: 1.8; CI: 1.1–2.9; *p* = 0.03), respectively (Figure 3). Patients with a VR > 1.64 were 2.2 times more likely to have an extended LOS in the ICU compared to those who had a VR < 1.64 (HR: 2.2; CI: 1.3–3.5; *p* < 0.01) (Figure 4A). There was no significant association between a VR > 1.64 and the LOS in the hospital (HR: 1.4; CI: 0.9139–2.261; *p* = 0.12) (Figure 4B). There was no significant association between a PFR < 276 and the LOS in the ICU (HR: 1.161; CI: 0.6842–1.969; *p* = 0.58 or in the hospital (HR: 0.8015; CI: 0.4949–1.298; *p* = 0.37) (Figure 4C,D).

## 4. Discussion

In this study, 83% of ARDS patients were successfully weaned from VV-ECMO. Patients who experienced unsafe liberation had significantly higher ventilatory ratios (VRs) and significantly lower P_a_O_2_/FiO_2_ ratios (PFRs) compared to those who were safely weaned from VV-ECMO. The ventilatory ratio was identified as an independently significant predictor of unsafe liberation, with higher VR values being associated with an increased risk of unsuccessful weaning. To achieve a sensitivity of >90%, a VR value of 1.64 was identified. Patients who were unsafely liberated from VV-ECMO experienced longer ICU and hospital stays. Patients with a VR value > 1.64 were associated with significantly longer ICU lengths of stay compared to those with a VR value < 1.64. Patients who underwent unsafe liberation from VV-ECMO did not exhibit significantly higher mortality compared to those who were safely weaned from VV-ECMO.

Decannulation from VV-ECMO signifies a critical phase in the management of patients with severe respiratory failure. It necessitates the comprehensive restoration of native lung function, ensuring that it can effectively meet the metabolic demands of the body, including oxygen uptake and carbon dioxide removal. This process is not only a matter of time for lung healing but also requires the support of ECMO, which plays a pivotal role in maintaining lung protection throughout this transitional period. Achieving the delicate balance between native lung recovery and ECMO support is essential for successful liberation from extracorporeal life support and optimizing patient outcomes.

ARDS alters oxygenation, ventilation, and compliance in patients. While oxygenation and compliance are routinely assessed in the management of ventilator support in ARDS, ventilation is rarely assessed. The PF ratio is a parameter used to assess the oxygenation of a patient [26]. It is commonly used to assess the severity of ARDS and is calculated by dividing the partial pressure of oxygen (PaO_2_) in the arterial blood by the fraction of inspired oxygen (FiO_2_) the patient is receiving. A lower PF ratio indicates impaired oxygenation. In contrast, the VR focuses on evaluating the efficiency of ventilation, and is closely correlated with dead space. The VR provides valuable insights into how effectively a patient is ventilating and eliminating carbon dioxide, providing information on respiratory efficiency rather than oxygenation status. Our study indicates that the VR, as an indicator of dead space, appears to be a more effective predictor of unsuccessful liberation from VV-ECMO than the PFR.

In line with our findings, Lazzari et al. [27] proposed that weaning failure from VV-ECMO comes from the increased effort required to eliminate CO_2_ rather than just to measure the oxygenation status of the patient. The authors showed, in a multicenter prospective and retrospective study cohort, that patients with a higher end-tidal to arterial partial carbon dioxide pressure ratio had a higher likelihood of successful weaning from VV-ECMO.

Al-Fares [7] conducted a retrospective study including 55 patients, where 21 out of 55 patients did not meet the criteria for safe liberation. The authors found that the VR was significantly higher in patients who were unsafely liberated from VV-ECMO. However, the VR only showed an imprecise association with unsafe liberation after adjusting for age and sequential organ failure assessment score. Instead, the results of this study revealed that both the tidal volume per predicted body weight (VT_pbw_) and heart rate (HR) recorded at the end of the sweep gas off trial (SGOT) independently correlated with an increased likelihood of unsafe liberation from VV-ECMO. While the prospective study cohort of this study confirms the association between tidal volume per predicted body weight (VT_pbw_) and HR with unsafe liberation from VV-ECMO, it also showed a significant association between higher inspiratory effort, as indicated by the esophageal pressure swings and unsafe liberation. The findings of this study emphasize the significance of considering multiple parameters rather than relying on a single factor when predicting safe or unsafe liberation from VV-ECMO.

In this study, during the ROC analysis, we determined a cutoff value for the VR of 1.64. At this cutoff value, a sensitivity of almost 93% and a specificity of 56% were observed. This means that in over 90% of the cases with a VR > 1.64, the patient will correctly not be weaned from the VV-ECMO. Conversely, it implies that if the VR < 1.64, there is a 56% probability that the patient is correctly identified as someone who can be weaned from the VV-ECMO.

In considering the clinical implications of VR cutoff values, our primary goal was to ensure a high sensitivity. This approach minimizes the risk of premature weaning from VV-ECMO, which can lead to urgent and complex re-interventions. Although this strategy reduces specificity, leading to some patients being maintained on VV-ECMO longer than necessary, the potential complications of premature weaning justify this trade-off. Thus, we determined that a VR cutoff value of 1.64, with a sensitivity of 93% and a specificity of 56%, would provide the best balance for safe patient management.

Additionally, we were unable to demonstrate a difference in mortality between patients with safe versus unsafe liberation. However, it should be mentioned that the *p*-value showed a trend toward significance, suggesting a possible impact of unsuccessful liberation from VV-ECMO on mortality (*p* = 0.05). A higher number of patients would very likely be able to demonstrate significance.

### Limitations

This study has several limitations. First, its retrospective design inherently exposes it to the potential biases and limitations associated with the analysis of historical patient data. Secondly, the study’s relatively small sample size may limit the generalizability of its findings. Implementing a prospective study design with a larger patient cohort would enhance the validity and reliability of the results. Moreover, the inclusion of data from only two medical centers in which weaning practices from VV-ECMO may differ introduces the possibility of center-specific biases. However, the data were collected based on the clinical parameters measured at a consistent time point: 30 min following the start of the SGOT. Furthermore, it is important to note that the calculation of the SOFA score was not possible for a subset of patients due to missing data from MGH. Despite this limitation, we believe that the absence of these data did not significantly impact the overall results of the study. However, a prospective multicenter study is needed to assess the clinical impact of the ventilatory ratio (VR) and further optimize the management of weaning from VV-ECMO. This critical step will not only provide more insights into the predictive power of the VR but also help refine our strategies for liberating patients from VV-ECMO. Finally, it is also highly possible that the small sample size contributed to the lack of a statistically significant difference in mortality between the safe and unsafe liberation groups.

It is possible that patients who are on VV-ECMO because of COVID-19 behave differently from patients on VV-ECMO from other forms of ARDS. However, the data suggest that ventilated patients with COVID-19-associated ARDS are best treated with the same lung-protective ventilation strategies that are recommended for non-COVID-19-associated ARDS [28]. Initial reports about distinct phenotypes of COVID-19 (H and L phenotypes, for example) [29] were not substantiated in further research. We, therefore, do not believe that patients on VV-ECMO for COVID-19-induced ARDS will behave differently when it comes to respiratory physiology than non-COVID-19 ARDS. It is also important to emphasize that the occurrence of COVID-19-induced ARDS was not statistically different between the two groups of this study (safe liberation vs. unsafe liberation).

## 5. Conclusions

In conclusion, our study suggests that the ventilatory ratio (VR) holds promise as a valuable predictor for safe liberation from VV-ECMO in ARDS patients, with higher VR values associated with an increased risk of unsuccessful weaning. It also emphasizes the complexity of weaning processes and highlights the need for a comprehensive approach to prediction and management. Larger randomized controlled trials are needed to better understand the impact of the VR on the success of weaning from VV-ECMO.

## Figures and Tables

**Table 1:** Baseline characteristics of patients with severe ARDS with safe or unsafe liberation from VV-ECMO.

	All Patients	Safe Liberation	Unsafe Liberation	*p*-Value
Characteristic	n = 84	n = 70	n = 14	
Age, y	51 (39–60)	51 (36–60)	51 (45–53)	0.89
Male sex	50 (60%)	39 (56%)	11 (79%)	0.14
BMI, kg/m^2^	51.1 (29.3–78.9)	58.8 (29.6–79.0)	31.5 (26.8–68.9)	0.31
Reason for ECMO				
ARDS due to COVID-19 pneumonia	30 (36%)	23 (33%)	7 (50%)	0.36
ARDS due to another cause of pneumonia	25 (30.5%)	22 (31%)	3 (21.5%)	0.54
ARDS post lung transplant	8 (9.5%)	8 (11%)	0 (0%)	0.34
Postoperative respiratory failure	5 (6%)	4 (6%)	1 (7%)	0.85
Other ^1^	16 (19%)	13 (19%)	3 (21.5%)	0.81
VV-ECMO duration, d	11 (5–20)	10 (4–19)	16 (13–19)	0.04 *
	All Patients	Safe Liberation	Unsafe Liberation	*p*-Value
Characteristic	n = 40	n = 31	n = 9	
SOFA score during SGOT ^3^	6 (3–7)	5 (3–7)	7 (6–9)	0.18

The results are expressed as the median (from the first quartile to the third quartile) or number (%). BMI: body mass index; VV-ECMO: veno-venous extracorporeal membrane oxygenation; SOFA: sequential organ failure assessment. Significant results are marked with an *. ^1^ Other reasons include cardiotomy, interstitial lung disease, bronchopleural fistula, pulmonary edema, status asthmaticus, tracheal rupture, ketoacidosis, and pulmonary arteriovenous malformation. ^3^ Due to missing values at MGH, the SOFA score calculation was only possible for patients treated at the University Hospital Aachen.

**Table 2:** VRs and PFRs of patients with severe ARDS with safe or unsafe liberation from VV-ECMO.

	All Patients n = 84	Safe Liberation n = 70	Unsafe Liberation n = 14	*p*-Value
Characteristic				
VR	1.65 (1.39–2.81)	1.58 (1.32–1.91)	1.89 (1.79–2.81)	<0.01 *
PFR	241 (164–276)	243 (185–287)	169 (110–212)	0.01 *

The results are expressed as a median (from the first quartile to the third quartile). Patients with unsafe liberation from VV-ECMO had a significantly higher VR compared to the patients who could be safely liberated from VV-ECMO. Patients with unsafe liberation from VV-ECMO showed a significantly lower PFR compared to the patients who could be safely liberated from VV-ECMO. VV-ECMO: veno-venous extracorporeal membrane oxygenation; VR: ventilatory ratio; PFR: P/F ratio (p_a_O_2_/FiO_2_). Significant results are marked with an *.

**Table 3:** Predictors of unsafe liberation from VV-ECMO for ARDS using multivariate logistic regression.

	OR (95%) CI	*p*-Value
Characteristic		
VR	0.38 (0.17–0.81)	0.01 *
PFR	1.00 (0.99–1.01)	0.35
Duration on VV-ECMO	1.00 (0.98–1.02)	0.83

The VR was the only parameter that was shown to be a predictor of unsafe liberation from VV-ECMO. VV-ECMO: veno-venous extracorporeal membrane oxygenation; VR: ventilatory ratio; PFR: P/F ratio (P_a_O_2_/FiO_2_). Significant results are marked with an *.

**Table 4:** ICU LOS, hospital LOS, and mortality in patients with safe and unsafe liberation from VV-ECMO.

	All Patients n = 84	Safe Liberation n = 70	Unsafe Liberation n = 14	*p*-Value
Characteristic				
Hospital LOS, days	41 (28–62)	38 (27–59)	61 (38–79)	0.04 *
ICU LOS, days	31 (17–51)	26 (16–45)	58 (30–78)	<0.01 *
Mortality, %	14 (17)	9 (13)	5 (38)	0.051

The results are based on a binary logistic regression. The ICU LOS and hospital LOS were significantly higher in patients unsafely liberated from VV-ECMO. There was no significant difference in mortality between patients with safe and unsafe liberation from VV-ECMO. LOS: length of stay; ICU: intensive care unit; VV-ECMO: veno-venous ECMO. Significant results are marked with an *.

## Data Availability

The datasets generated and/or analyzed during the current study are available from the corresponding author upon reasonable request.

## Supplementary Materials

The following are available online at https://www.mdpi.com/article/10.3390/jcm13133758/s1.

## Appendix A

### Appendix A.1. Standard Operating Procedure for Weaning from VV-ECMO

#### Appendix A.1.1. University Hospital Aachen

Weaning can be initiated once pulmonary stabilization has been achieved. When blood flow has been successfully reduced to approximately 2 L per minute, lung-protective ventilation settings are applied. Subsequently, both blood and gas flows are adjusted to 1 L per minute. If the pCO_2_ levels and SaO_2_ remain stable, gas flow is stopped, and blood gas analysis is withdrawn after 1 h. If the values are stable, heparinization is discontinued, and 2 h later, the VV-ECMO is removed. After the clamped cannulas are removed, manual compression is applied until hemostasis is achieved, followed by a compression bandage for 4 h.

#### Appendix A.1.2. Massachusetts General Hospital

Standard ventilator settings on VV-ECMO at the Massachusetts General Hospital are pressure control/assist control − driving pressure 10 cm H_2_O, optimal PEEP, and FiO_2_ 0.40 or less. Once the patient’s tidal volume on the above settings is >2 mL/kg IBW, a recruitment maneuver/best PEEP trial is performed to optimize lung volumes. Once tidal volumes are > 4 mL/kg IBW, the patient is transitioned to the volume control/assist control settings with optimal PEEP and lung protective parameters to achieve a driving pressure of ≤10 cm H_2_O (higher driving pressures may be tolerated if there are circuit complications). The respiratory rate on the ventilator is increased, and the sweep gas is reduced while monitoring for normocapnia. The sweep gas is then turned off for 12–24 h while monitoring the patient’s gas exchange and lung mechanics (the SGOT or “cap” trial). Of note, the flow in the VV-ECMO circuit is not reduced at MGH. If the patient passes the SGOT, anticoagulation is held, and the cannulas are removed at the bedside with a figure-of-eight suture placed around the cannula together with manual pressure for hemostasis.

## References

[1] Tonna J.E., Abrams D., Brodie D., Greenwood J.C., Rubio Mateo-Sidron J.A., Usman A., Fan E. (2021). Management of Adult Patients Supported with Venovenous Extracorporeal Membrane Oxygenation (VV ECMO): Guideline from the Extracorporeal Life Support Organization (ELSO). ASAIO J..

[2] Quintel M., Bartlett R.H., Grocott M.P., Combes A., Ranieri M.V., Baiocchi M., Nava S., Brodie D., Camporota L., Vasques F. (2020). Extracorporeal Membrane Oxygenation for Respiratory Failure. Anesthesiology.

[3] Broman L.M., Malfertheiner M.V., Montisci A., Pappalardo F. (2018). Weaning from veno-venous extracorporeal membrane oxygenation: How I do it. J. Thorac. Dis..

[4] Gattinoni L., Vassalli F., Romitti F., Vasques F., Pasticci I., Duscio E., Quintel M. (2019). Extracorporeal gas exchange: When to start and how to end?. Crit. Care.

[5] Combes A., Hajage D., Capellier G., Demoule A., Lavoué S., Guervilly C., Da Silva D., Zafrani L., Tirot P., Veber B. (2018). Extracorporeal Membrane Oxygenation for Severe Acute Respiratory Distress Syndrome. N. Engl. J. Med..

[6] Grant A.A., Hart V.J., Lineen E.B., Badiye A., Byers P.M., Patel A., Vianna R., Koerner M.M., El Banayosy A., Loebe M. (2018). A Weaning Protocol for Venovenous Extracorporeal Membrane Oxygenation with a Review of the Literature. Artif. Organs.

[7] Al-Fares A.A., Ferguson N.D., Ma J., Cypel M., Keshavjee S., Fan E., Del Sorbo L. (2021). Achieving Safe Liberation During Weaning From VV-ECMO in Patients with Severe ARDS: The Role of Tidal Volume and Inspiratory Effort. Chest.

[8] Gannon W.D., Stokes J.W., Bloom S., Sherrill W., Bacchetta M., Rice T.W., Semler M.W., Casey J.D. (2021). Safety and Feasibility of a Protocolized Daily Assessment of Readiness for Liberation from Venovenous Extracorporeal Membrane Oxygenation. Chest.

[9] Reeb J., Olland A., Pottecher J., Delabranche X., Schaeffer M., Renaud S., Santelmo N., Kessler R., Massard G., Falcoz P.-E. (2017). Extracorporeal Membrane Oxygenation for Acute Respiratory Distress Syndrome After Pneumonectomy. Ann. Thorac. Surg..

[10] Li X., Guo Z., Li B., Zhang X., Tian R., Wu W., Zhang Z., Lu Y., Chen N., Clifford S.P. (2020). Extracorporeal Membrane Oxygenation for Coronavirus Disease 2019 in Shanghai, China. ASAIO J..

[11] Chaves R.C.D.F., Rabello R., Timenetsky K.T., Moreira F.T., Vilanova L.C.D.S., Bravim B.D.A., Serpa A., Corrêa T.D. (2019). Extracorporeal membrane oxygenation: A literature review. Rev. Bras. Ter. Intensiva.

[12] Seiler F., Trudzinski F.C., Hörsch S.I., Kamp A., Metz C., Flaig M., Alqudrah M., Wehrfritz H., Kredel M., Muellenbach R.M. (2018). Weaning from prolonged veno-venous extracorporeal membrane oxygenation (ECMO) after transfer to a specialized center: A retrospective study. J. Artif. Organs.

[13] Sen A., Callisen H.E., Alwardt C.M., Larson J.S., Lowell A.A., Libricz S.L., Tarwade P., Patel B.M., Ramakrishna H. (2016). Adult venovenous extracorporeal membrane oxygenation for severe respiratory failure: Current status and future perspectives. Ann. Card. Anaesth..

[14] Vasques F., Romitti F., Gattinoni L., Camporota L. (2019). How I wean patients from veno-venous extra-corporeal membrane oxygenation. Crit. Care.

[15] Teijeiro-Paradis R., Tiwari P., Spriel A., Del Sorbo L., Fan E. (2021). Standardized liberation trials in patients with COVID-19 ARDS treated with venovenous extracorporeal membrane oxygenation: When ready, let them breathe!. Intensive Care Med..

[16] Belliato M., Cremascoli L., Epis F., Ferrari F., Quattrone M.G., Malfertheiner M.V., Broman L.M., Aliberti A., Taccone F.S., Iotti G.A. (2021). Carbon Dioxide Elimination During Veno-Venous Extracorporeal Membrane Oxygenation Weaning: A Pilot Study. ASAIO J..

[17] Nuckton T.J., Alonso J.A., Kallet R.H., Daniel B.M., Pittet J.-F., E Isner M.D., Matthay M.A. (2002). Pulmonary Dead-Space Fraction as a Risk Factor for Death in the Acute Respiratory Distress Syndrome. N. Engl. J. Med..

[18] Ferluga M., Lucangelo U., Blanch L. (2018). Dead space in acute respiratory distress syndrome. Ann. Transl. Med..

[19] Doorduin J., Nollet J.L., Vugts M.P., Roesthuis L.H., Akankan F., van der Hoeven J.G., van Hees H.W., Heunks L.M. (2016). Assessment of dead-space ventilation in patients with acute respiratory distress syndrome: A prospective observational study. Crit. Care.

[20] Jayasimhan D., Chieng J., Kolbe J., Sidebotham D.A. (2023). Dead-Space Ventilation Indices and Mortality in Acute Respiratory Distress Syndrome: A Systematic Review and Meta-Analysis. Crit. Care Med..

[21] Sinha P., Fauvel N.J., Singh S., Soni N. (2009). Ventilatory ratio: A simple bedside measure of ventilation. Br. J. Anaesth..

[22] Sinha P., Calfee C.S., Beitler J.R., Soni N., Ho K., Matthay M.A., Kallet R.H. (2019). Physiologic analysis and clinical performance of the ventilatory ratio in acute respiratory distress syndrome. Am. J. Respir. Crit. Care Med..

[23] Torres A., Motos A., Riera J., Fernández-Barat L., Ceccato A., Pérez-Arnal R., García-Gasulla D., Peñuelas O., Lorente J.A., Rodriguez A. (2021). The evolution of the ventilatory ratio is a prognostic factor in mechanically ventilated COVID-19 ARDS patients. Crit. Care.

[24] Maj R., Palermo P., Gattarello S., Brusatori S., D’Albo R., Zinnato C., Velati M., Romitti F., Busana M., Wieditz J. (2023). Ventilatory ratio, dead space, and venous admixture in patients with acute respiratory distress syndrome. Br. J. Anaesth..

[25] NHLBI ARDS Network|Tools. http://www.ardsnet.org/tools.shtml.

[26] Bone R.C., Maunder R., Slotman G., Silverman H., Hyers T.M., Kerstein M.D., Ursprung J.J. (1989). An early test of survival in patients with the adult respiratory distress syndrome. The PaO2/FIo2 ratio and its differential response to conventional therapy. Prostaglandin E1 Study Group. Chest.

[27] Lazzari S., Romitti F., Busana M., Vassalli F., Bonifazi M., Macrí M.M., Giosa L., Collino F., Heise D., Golinski M. (2022). End-Tidal to Arterial Pco2 Ratio as Guide to Weaning from Venovenous Extracorporeal Membrane Oxygenation. Am. J. Respir. Crit. Care Med..

[28] Dragoi L., Siuba M.T., Fan E. (2023). Lessons Learned in Mechanical Ventilation/Oxygen Support in Coronavirus Disease 2019. Clin. Chest Med..

[29] Gattinoni L., Chiumello D., Caironi P., Busana M., Romitti F., Brazzi L., Camporota L. (2020). COVID-19 pneumonia: Different respiratory treatments for different phenotypes?. Intensive Care Med..

