# Supplementary material for: The Ventilatory Ratio as a Predictor of Successful Weaning from a Veno-Venous Extracorporeal Membrane Oxygenator
Source: J Clin Med. 2024 Jun 27;13(13):3758. doi: 10.3390/jcm13133758 (PMC11242634; doi:10.3390/jcm13133758)
Supplement: Supplementary file 1 [file jcm-13-03758-s001.zip › jcm-2981374-supplementary.pdf]

```
LOGISTIC REGRESSION VARIABLES Success
  /METHOD=ENTER VRratio PFR Durationdays
  /CLASSPLOT
  /PRINT=CI(95)
  /CRITERIA=PIN(0.05) POUT(0.10) ITERATE(20) CUT(0.5).
```

Logistische Regression

Hinweise

Ausgabe erstellt		08-FEB-2024 18:44:31
Kommentare		
Eingabe	Aktives Dataset	DataSet1
	Filter	<ohne>
	Stärke	<ohne>
	Aufgeteilte Datei	<ohne>
	Anzahl Zeilen in Arbeitsdatendatei	84
Handhabung fehlender Werte	Definition von 'Fehlend'	Benutzerdefiniert fehlende Werte werden als fehlend behandelt
Syntax		LOGISTIC REGRESSION VARIABLES Success<br>/METHOD=ENTER VRratio PFR Durationdays<br>/CLASSPLOT<br>/PRINT=CI(95)<br>/CRITERIA=PIN(0.05) POUT(0.10) ITERATE(20) CUT(0.5).
Ressourcen	Prozessorzeit	00:00:00,02
	Verstrichene Zeit	00:00:00,01

Zusammenfassung der Fallverarbeitung

Ungewichtete Fälle[a]		H	Prozent
Ausgewählte Fälle	Einbezogen in Analyse	83	98,8
	Fehlende Fälle	1	1,2
	Gesamtsumme	84	100,0
Nicht ausgewählte Fälle		0	,0
Gesamtsumme		84	100,0

a. Wenn die Gewichtung in Kraft ist, finden Sie in der Klassifikationstabelle die Gesamtzahl von Fällen.

Codierung abhängiger Variablen

Ursprünglicher Wert	Interner Wert
FAIL	0
Erfolgreich	1

Block 0: Anfangsblock

Klassifikationstabelle[a,b]

Beobachtet			Vorhersagewert		
			Success		Prozentsatz richtig
			FAIL	Erfolgreich	
Schritt 0	Success	FAIL	0	14	,0
		Erfolgreich	0	69	100,0
	Gesamtprozentsatz				83,1

a. Die Konstante ist im Modell enthalten.

b. Der Trennwert ist ,500

Variablen in der Gleichung

		B	Standardfehler	Wald	df	Sig.	Exp(B)
Schritt 0	Konstante	1,595	,293	29,611	1	,000	4,929

Nicht in der Gleichung vorhandene Variablen

			Score	df	Sig.
Schritt 0	Variablen	VRratio	9,181	1	,002
		PFR	2,235	1	,135
		Durationdays	,024	1	,877
	Gesamtstatistik		10,210	3	,017

Block 1: Methode = Eingabe

Omnibustests der Modellkoeffizienten

		Chi-Quadrat	df	Sig.
Schritt 1	Schritt	9,196	3	,027
	Block	9,196	3	,027
	Modell	9,196	3	,027

Modellübersicht

Schritt	-2 Log-Likelihood	R-Quadrat nach Cox & Snell	R-Quadrat nach Nagelkerke
1	66,131[a]	,105	,176

a. Die Schätzung wurde bei Iteration Nummer 5 beendet, da Parameterschätzungen sich um weniger als ,001 geändert haben.

Klassifikationstabelle[a]

Beobachtet			Vorhersagewert		
			Success		Prozentsatz richtig
			FAIL	Erfolgreich	
Schritt 1	Success	FAIL	3	11	21,4
		Erfolgreich	0	69	100,0
	Gesamtprozentsatz				86,7

a. Der Trennwert ist ,500

Variablen in der Gleichung

		B	Standardfehler	Wald	df	Sig.	Exp(B)
Schritt 1[a]	VRratio	-,979	,393	6,201	1	,013	,376
	PFR	,004	,004	,869	1	,351	1,004
	Durationdays	,002	,010	,046	1	,830	1,002
	Konstante	2,671	1,299	4,225	1	,040	14,452

Variablen in der Gleichung

		95% Konfidenzintervall für EXP (B)	
		Unterer	Oberer
Schritt 1[a]	VRratio	,174	,812
	PFR	,996	1,011
	Durationdays	,983	1,022
	Konstante		

a. In Schritt 1 eingegebene Variable(n): VRratio, PFR, Durationdays.

```
Step number: 1
```

```
                  Observed Groups and Predicted Probabilities

      16 +
                                                  +
         I
                                                  I
         I
                                                  I
F        I
                                                  I
R     12 +
                                                  +
E        I
                                                  I
Q        I
                                                  I
U        I
                             E  E        I
E      8 +
                             E  E        +
N        I
                        E  E E  E        I
C        I
                        E  E EE E   E    I
Y        I
                        E  E EE E   E    I
       4 +
                        E  E EE E E E    +
         I
           E    EE EEEE E E E    I
         I                                                   EE
      E    FEEE EEEFEEE E EEEE   I
         I                          F              F  F      EE      E      F
 E   FE    FFFE FEEFEEEFE EEFE   I
Predicted ---------+---------+---------+---------+---------+---------+-------
--+---------+---------+---------
  Prob:   0       ,1        ,2        ,3        ,4        ,5        ,6
 ,7        ,8        ,9         1
  Group:  FFFFFFFFFFFFFFFFFFFFFFFFFFFFFFFFFFFFFFFFFFFFFFFFFFEEEEEEEEEEEEEEEEE
EEEEEEEEEEEEEEEEEEEEEEEEEEEEEEEEE

          Predicted Probability is of Membership for Erfolgreich
```

```
The Cut Value is ,50
Symbols: F - FAIL
         E - Erfolgreich
Each Symbol Represents 1 Case.
```
